# Prevalence of Cigarette Advertising and Other Promotional Strategies at the Point of Sale in St Louis, Missouri: Analysis by Store Type and Distance From a School

**DOI:** 10.5888/pcd11.130150

**Published:** 2014-04-17

**Authors:** Joaquin Barnoya, Graham Colditz, Sarah Moreland-Russell, Julianne Cyr, Doneisha Snider, Mario Schootman

**Affiliations:** Authors Affiliations: Graham Colditz, Sarah Moreland-Russell, Julianne Cyr, Doneisha Snider, Mario Schootman, Washington University in St Louis, St Louis, Missouri.

## Abstract

**Introduction:**

Point-of-sale advertising provides an opportunity for the tobacco industry to communicate with current and potential smokers. The US Family Smoking Prevention and Tobacco Control Act allows states to implement policies requiring that tobacco products be placed out of sight, and the Food and Drug Administration is considering banning point-of-sale advertising within 1,000 feet of schools. Our objective was to compare cigarette point-of-sale advertising near schools with grades prekindergarten through 12 and by store type.

**Methods:**

All registered cigarette retailers (n = 1,229) and schools (n = 581) in the city of St Louis and St Louis County were geocoded and mapped by using ArcGIS. Retailers were divided into 2 groups, those within 1,000 feet and those within 1,001 to 2,000 feet of a school; 200 retailers from each group were randomly selected. We assessed tobacco interior and exterior advertising, brands advertised, discounts, gifts with purchase, “no sales to minors” signage, and cigarette functional items (eg, advertising on shopping baskets). Analyses were done by distance from a school and store type.

**Results:**

We analyzed 340 retailers. Most retailers within 1,000 feet (91.2%) and from 1,001 to 2,000 feet (94.2%) of a school displayed cigarette advertising (*P* = .20). Convenience stores had the highest number of interior ads. In multivariable models, distance from school explained 0.2% of the variance in total advertising.

**Conclusion:**

Cigarette point-of-sale advertising is highly prevalent in St Louis within 1,000 feet of schools. A ban based on distance from a school might decrease advertising exposure, but its effect on smoking prevalence is yet to be determined because advertising farther from schools would still prevail.

## Introduction

Tobacco industry marketing campaigns have been instrumental in spreading the tobacco use epidemic worldwide. The industry has used several channels to market tobacco products, including television, magazines, billboards, radio, and sponsorships (eg, sporting and cultural events) (1). As restrictions on advertising become more widespread, the industry is focusing on the point of sale as their main advertising channel (2). In 2010 the industry spent almost $8.05 billion on advertising and promotion; price discounts paid to cigarette retailers to reduce the price of cigarettes to consumers accounted for almost 81% ($6.49 billion) (3). Although spending on point-of-sale advertising has declined in recent years, it was $163.7 million in 2008 (3). These advertising strategies are all effective ways to reach customers. Tobacco point-of-sale advertising influences adolescents’ smoking behaviors (4,5). Having a favorite cigarette advertisement and owning or being willing to use a cigarette promotional item increase the odds of future adult smoking (6). Furthermore, advertising increases the odds of trying smoking, and promotional items support the transition from an experimenter to an established smoker (7,8).

In 2009, the Family Smoking Prevention and Tobacco Control Act (FSPTCA) was passed in the United States. This act grants the Food and Drug Administration (FDA) specific regulatory authority to restrict the sale, distribution, accessibility, advertising, and promotion of tobacco products “consistent with and to the full extent permitted by the first amendment to the Constitution” (9). Furthermore, the Act grants state and local governments new authority to restrict the time, place, and manner of cigarette advertising. Specifically, it allows states to enact point-of-sale legislation that requires that tobacco products be kept behind the counter or out of sight and could potentially prohibit retail tobacco sales near elementary or secondary schools. The aim of this distance limit is to protect school students from exposure to tobacco advertising (4). As of October 2013, to the best of our knowledge no state or local government had taken full advantage of the FSPTCA to restrict advertising (Rhode Island and New York City have only banned price promotions). Although the FDA is debating just how far from schools to place the advertising restrictions (eg, 350 ft, 500 ft, or 1,000 ft), it has been shown that a limit of at least 1,000 feet is needed for the policy to be effective in reducing exposure (10).

Among the 50 states, Missouri ranks 38th in adolescent (aged 12–17 years old) smoking prevalence (11.8% compared with 10.1% nationally) (11). Therefore, the FSPTCA is particularly relevant to Missouri where there are no state-wide advertising restrictions at the point of sale. The objective of our study was to document the prevalence of cigarette point-of-sale advertising and to assess whether the FSPTCA would significantly affect cigarette advertising at the point of sale near schools in the St Louis City and County.

## Methods

We obtained a list of all registered retailers licensed to sell tobacco from the Missouri Department of Mental Health’s Division of Alcohol and Drug Abuse and a list of all schools with grades prekindergarten through 12 (charter, public, and private) from the Missouri Department of Education. Tobacco retailers (n = 1,229) and schools (n = 581) in St Louis City and County were then mapped in ArcGIS 10 (ESRI, Inc., Redlands, California). This is a suite of geographic information system software for working with maps and geographic information. Following geocoding of the retailers and school street addresses, we conducted a proximity analysis using ArcGIS to determine which retailers were within 1,000 feet of a school and which were within 1,001 to 2,000 feet of a school. Euclidean distance (straight line), as opposed to network distance (using the road network), was used to calculate distance between a school and a tobacco retailer. We chose the former, given that the latter will measure vehicular travel distance rather than walking distance, because a significant proportion of students walk to school (12). Regardless, Euclidean and actual distances measured by streets are highly correlated (>.90) (13). The distance range was chosen given the FDA’s debate regarding policy on distance from schools and point-of-sale advertising. Of the total retailers, 296 (24%) were within 1,000 feet of a school. A sensitivity analysis was done by using a distance limit of 350 feet to take into consideration if a shorter distance range was chosen by the FDA (14). By using a random digit generator, 200 retailers were selected from each distance group by using simple random sampling. Stores were visited between December 2009 and February 2010 by 2 trained research assistants. Of the 400 selected stores, 340 (85.5%) were included in the analysis. Retailers were excluded (n = 59, 17%) if the store was closed or could not be located and if it sold cigarettes only through a vending machine, sold cigars but no cigarettes, if no one under 18 was allowed in the store, and if the retailer was part of the instrument pilot testing or had incomplete data (n = 2, 0.5%). Small grocery stores, supermarkets, convenience stores (with and without gas stations), drugstores, liquor stores, and other store types (eg, tobacco retailers, restaurants, delicatessens) were surveyed. Although children are unlikely to visit liquor stores, we included all stores in recognizing that point of sale also influences young adults and current smokers (15).

To assess the prevalence and characteristics of point-of-sale advertising in stores, we trained auditors to use a checklist developed by Feighery et al (16). The checklist includes basic store information (eg, number of cash registers as a proxy for store size) and assesses interior and exterior advertising, brands advertised, discounts, gifts with purchase, “no sales to minors” signage, and cigarette functional items (eg, advertising on shopping baskets, clocks). In addition, the proximity of cigarette advertising to candy was recorded.

Analyses were performed by using descriptive (percentages, means, and standard deviation [SD]), nonparametric (χ^2^ and Mann–Whitney U test), and parametric statistics (*t* test and analysis of variance). Multiple linear regression was used to estimate total (exterior plus interior) and interior number of ads as continuous dependent variables. Predictor variables (store type, number of cashiers, price discounts, and distance from a school) were chosen based on the previously identified predictors of point-of-sale advertising (17,18). We used PASW 18.0 (SPSS Inc, Chicago, Illinois) to enter and analyze data.

## Results

Of the 340 stores sampled (171 within 1,000 ft and 169 within 1,001–2,000 ft of a school), most were convenience stores with gas stations, followed by small, independently owned grocery stores (small markets) (Table 1). Nearly all (92.7%) retailers displayed at least 1 cigarette advertisement, and most of these were inside the store. There was no significant difference in displaying at least 1 cigarette advertisement between retailers within 1,000 feet (91.2%) and those from 1,001 to 2,000 feet (94.2%) of a school (*P* = .20). Several retailers displayed advertisements (12.3%) or cigarette products (1.5%) near (within 50 cm of) candy. Most retailers (72.8%) had a promotional sale price, and 21.1% offered multi-pack discounts. A gift with cigarette purchase was found in 2.3% of the stores. Most (88.6%) stores posted a “no sales to minors” sign.

**Table 1:** Retail Stores (N = 340) Classified by Type and Distance From a School, St Louis, Missouri, 2010

Store Type	≤1,000 ft (n = 171), n (%)	1,001–2,000 ft^a^ (n = 169), n (%)
Supermarket	18 (10.5)	17 (10.5)
Small independently owned grocery stores (small markets)	46 (26.9)	45 (26.6)
Convenience store without gas station	8 (4.7)	4 (2.4)
Convenience store with gas station	68 (39.8)	53 (31.4)
Drugstore	18 (10.5)	21 (12.4)
Liquor store	6 (3.5)	14 (8.3)
Other	7 (4.1)	15 (8.9)

a *P* = .14 for difference between all store types and distance from a school.

When analyzed by distance from a school (Table 2), there was no significant difference in advertising, special pricing, multi-pack discounts, or “no sales to minors” signage. The difference in percentage of stores that had any exterior ads was not significantly (*P* = .50) different between stores within 1,000 feet (64%) and those within 1,001 to 2,000 feet (60%). In a sensitivity analysis we used 350 feet as the distance cutoff point; however, results remained unchanged (no difference in interior and exterior advertising). The total number of stores that had 13 (the total mean number) or more ads was not significantly (*P* = .50) different from the number of stores within 1,000 feet (n = 81, 47.4%) and those within 1,001 to 2,000 feet (n = 86, 50.3%) of a school. Furthermore, multiple regression models showed that distance from a school is not a significant predictor (*P* > .05) of point-of-sale advertising and explained 0.2% of the variance of total number of ads and 0.6% of the variance of total number of interior ads. When number of cash registers and store type were included in the analyses, the model explained 11.3% of the variance in the total number of ads and 6.6% of the variance of interior ads. Excluding supermarkets and liquor stores, where youth might likely spend less time, yielded the same results.

**Table 2:** Tobacco Point-of-Sale Advertising and Marketing in Retail Stores by Distance From a School, St Louis, Missouri, 2010

Characteristic	Median (25th–75th Centile) for Retail Stores Within 1,000 ft (n = 171)	Median (25th–75th Centile) for Retail Stores Within 1,001–2,000 ft (n = 169)	*P* value^a^
Cash registers	2 (1–2)	2 (1–3)	.80
Cigarette ads	12 (6–19)	13 (5–18)	.70
Interior ads	9 (4–14)	10 (5–14)	.50
Exterior ads	2 (0–5)	2(0–4)	.50
Special pricing	76.6 (131)	69.0 (118)	.10
Multi-pack discounts	21.1 (36)	21.1 (36)	.90
“No sales to minors” signage	86.5 (148)	85.4 (146)	.60

a *P* value calculated with Mann–Whitney U test for variables with the median and interquartile range and with χ^2^ test for proportions.

Regarding type, mean number of total cigarette ads was highest in liquor stores (16.8 [10.9] ads) and convenience stores with (15.1 [9.1] ads) and without (14.9 [7.8] ads) a gas station compared with other store types (Table 3). More than half of convenience stores without (66.7%) or with (61.5%) a gas station, small markets (62.6%), and liquor stores (55%) had at or above the mean total number, 13, of ads (Figure). Supermarkets (11.4%), drugstores (12.5%), and other store types (31.8%) were less likely to have more than the mean total number of ads. The percentage of stores that had any exterior ads was different (*P* < .001) by store type. Most convenience stores with (86%) and without (67%) a gas station, liquor stores (85%), and small markets (77%) had exterior ads. Exterior ads were less likely to be found in other store types (37%), supermarkets (11%), and drugstores. Promotional sales pricing were most frequently found in convenience stores with no gas station, followed by convenience stores with gas stations, drugstores, small markets, liquor stores, and supermarkets (Table 3).

**Table 3:** Tobacco Retailers’ Point-of-Sale Advertising by Store Type, St Louis, Missouri, 2010

Characteristic	Total (N = 340)	Small Market (n = 91)	Supermarket (n = 35)	Convenience Store With Gas Station (n = 121)	Convenience Store Without Gas Station (n = 12)	Drugstore (n = 39)	Liquor Store (n = 20)	Other (n = 22)	*P* Value
Cash registers, mean (SD)	3.1 (3.8)	1.4 (0.6)	11.9 (5.5)	1.6 (0.7)	2.0 (0.0)	5.8 (1.7)	1.5 (0.7)	1.1 (0.3)	<.001
Total cigarette ads,^a^ mean (SD)	13.0 (10.1)	14.2 (9.0)	6.9 (5.6)	15.1 (9.1)	14.9 (7.8)	7.2 (4.4)	16.8 (10.9)	12.7 (20.8)	<.001
Interior ads, mean (SD)	10.1 (7.8)	10.6 (6.9)	6.5 (5.2)	10.8 (6.7)	13.0 (6.4)	7.1 (4.4)	12.8 (8.3)	10.4 (17.2)	.04
Special pricing, % (n)	72.8 (249)	74.7 (68)	65.7 (23)	78.7 (96)	91.7 (11)	77.5 (31)	70.0 (14)	27.3 (6)	<.001
Multi-pack discount, % (n)	21.1 (72)	5.5 (5)	5.7 (2)	20.5 (25)	66.7 (8)	75.0 (30)	5.0 (1)	4.5 (1)	<.001
“No sales to minors” signage, % (n)	88.6 (303)	75.8 (69)	94.3 (33)	97.5 (119)	100 (12)	95.0 (38)	100 (20)	54.5 (12)	<.001

Abbreviation: SD, standard deviation.

a Includes interior and exterior ads.

**Figure:**
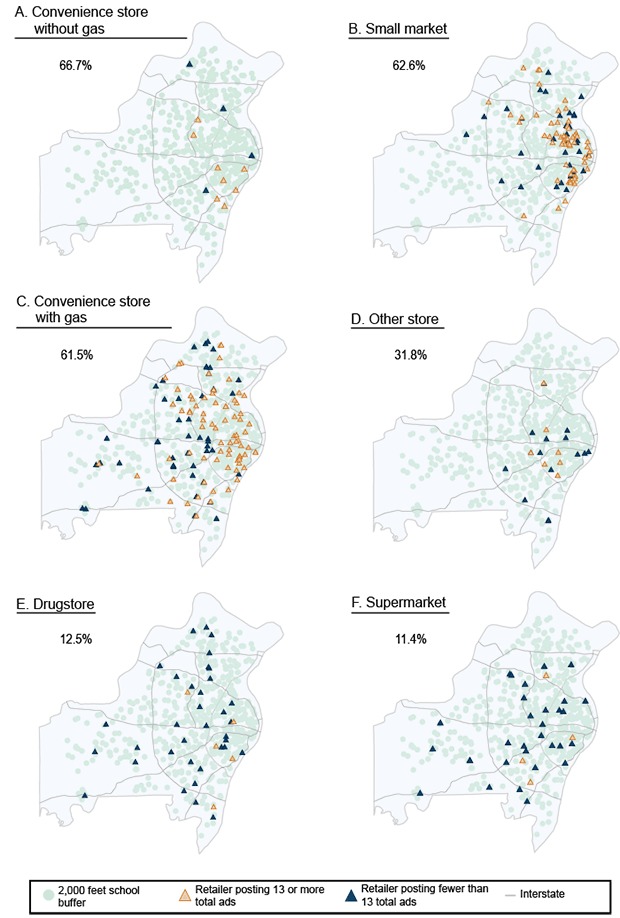
Cigarette point-of-sale advertising in tobacco retailers by store type and distance from a school in St Louis, Missouri, 2010. Percentages shown are the percentage of stores posting the mean number (13) of total ads or more in St Louis City and County.

## Discussion

Tobacco point-of-sale advertising is highly prevalent in St Louis. The prevalence of advertising in stores is independent of distance from a school but dependent on store type. Furthermore, prominent pack display and multi-pack and promotional discounts are also highly prevalent.

Point-of-sale advertising (including prominent pack display) has been for the most part ignored in tobacco control programs worldwide. For example, except for Scotland and Ireland, none of the advertising bans implemented in the world’s largest cigarette markets (Australia, Brazil, Canada, China, India, Indonesia, Japan, Russia, and the United States) include a comprehensive ban at the point of sale (19,20). In Hong Kong, the effect of an advertising ban to decrease brand recognition among children was less successful because the point of sale (and sponsorship) was not included (21). As a result, it is estimated that only 6% of the world’s population benefit from a comprehensive advertising ban (20).

Among adolescents, cigarette point-of-sale advertising increases students’ perception of the ease of purchasing cigarettes and decreases their likelihood for showing proof of age (22). Although not yet conclusive, point-of-sale advertising is associated with increased smoking prevalence and brand recognition (4). In adults, point-of-sale advertising increases brand recognition, makes it harder to quit, and increases smoking relapse in former smokers (15,16,23–25). Furthermore, in young adults it has been found to increase unplanned cigarette purchases (15). Therefore, even though the proposed ban by distance from a school aims to decrease youth exposure to cigarette advertising, the benefits of a comprehensive ban (regardless of distance from a school) would likely benefit others also influenced at the point of sale.

Our finding of promotional sales prices and multi-pack discounts is in agreement with the reported priorities of cigarette industry spending on promotional advertising (3,5,20). Since the early 1980s, the industry recognized the relevance of price incentives in particular for young adult smokers “interested in price” (26). As a result, by the early 1990s, discount brands accounted for almost 40% of the cigarette market, were more likely to be used by young adult smokers, and contributed to slowing the decline in smoking prevalence observed in the 1980s and the 1990s (26,27). The multipack discounting strategy has been used by the industry in response to tax increases, recognizing that these “closely targeted pack promotions in selected sites could lead to brand loyalty from repeated trial” (26). Therefore, these forms of price promotion should be banned according to the Framework Convention on Tobacco Control (28). However, most countries (60%) lack a comprehensive ban on promotional discounts (20). As of October 2013, only Brazil, Canada, India, Japan, and the United Kingdom had implemented a ban on promotional discounts (20). The point of sale should not be viewed as a classic advertising venue for the industry but rather a useful tool to reach potential young adults at risk of becoming smokers and to deter quitters.

Our data suggest that banning advertising based on distance from schools may decrease exposure of school-aged children and adolescents to point-of-sale cigarette advertising. However, even if we assume that it is near their schools where adolescents spend most of their spare time, it would ignore other issues with cigarette advertising. First, adolescents attending schools are not the only people influenced by point-of-sale advertising. Former and current smokers attempting to remain or become committed to quitting smoking are also negatively impacted by point-of-sale advertising (23). Second, as our results indicate, advertising is largely determined by store type as opposed to distance from a school as number of ads varied significantly across store types. When distance from school is held constant, the proportion of retailers with more than the mean number of ads by store type is highest in convenience stores with and without gas and small markets and lowest in supermarkets, drugstores, and other stores. In addition, the proposed 1,000 feet distance from a school is likely to miss most tobacco retailers (in our sample only 24% of them are within this range). If the range within which the ban would be effective is decreased (from 1,000 ft to 350 ft), the percentage of retailers included in the ban would be even lower (10).

Our data have several strengths and limitations. This is the first study to document cigarette point-of-sale advertising in St Louis and to assess its relationship with distance from a school (as proposed by the FSPTCA). However, the FDA includes both schools and playgrounds in their proposal. Our study does not include information on distance from a playground because we were not able to obtain playground location data. One solution was to use park location data; however, not all parks contain playgrounds. Although we did not include playgrounds in our analysis, our study is still relevant in that it focuses on schools, a place where children spend a large portion of their day. Furthermore, cigarette point-of-sale advertising has been found to be more prevalent in neighborhoods with a higher percentage of African Americans and of residents of low socioeconomic status (29,30). Therefore, the interaction between neighborhood ethnic distribution, socioeconomic status, school environment, and cigarette point-of-sale advertising merits additional research. Finally, even though our study did not assess the effect of advertising on smoking prevalence, our results do yield a high prevalence of point-of-sale advertising and therefore support the implementation of a comprehensive advertising ban.

To achieve its goal to decrease exposure to cigarette advertising overall (not only among adolescents around schools), the FSPTCA in the United States (and any other legislative body with the same objective) should include a ban at the point of sale in retail stores regardless of location and type. Furthermore, other advertising strategies implemented at the point of sale (eg, promotional discounts) should also be included in a comprehensive ban. Because point-of-sale advertising is widely integrated throughout the society, a comprehensive ban is needed to decrease adolescents’ exposure to tobacco advertising and other tobacco industry marketing strategies (eg, multiple packs and price discounts) that lead to smoking initiation and uptake. More than free speech, point-of-sale advertising represents a clear marketing strategy aimed at maintaining the public visibility and social acceptability of tobacco products, encouraging brand loyalty and further consumption among current smokers, and recruiting new smokers or former smokers. Despite what we know about the effects of point-of-sale advertising on adolescents, young adults, adults, and former smokers, further research is likely to support the notion that this type of advertising is inherently harmful to public health because it encourages smoking in several ways. Therefore, our data should aid tobacco control advocates to argue for a comprehensive advertising ban and serve as baseline to track FSPTCA implementation.
